# Dry-milled flour rice ‘Seolgaeng’ harbors a mutated fructose-6-phosphate 2-kinase/fructose-2,6-bisphosphatase2

**DOI:** 10.3389/fpls.2023.1231914

**Published:** 2023-08-10

**Authors:** Ju-Won Kang, Sang-Kyu Lee, Su-Hyeon Shim, Dongjin Shin, Jun-Hyeon Cho, Ji-Yoon Lee, Jong-min Ko, Hyeonso Ji, Hyang-Mi Park, Eok-Keun Ahn, Jong-Hee Lee, Jong-Seong Jeon

**Affiliations:** ^1^ Department of Southern Area Crop Science, National Institute of Crop Science, Rural Development Administration, Miryang, Republic of Korea; ^2^ Graduate School of Green-Bio Science and Crop Biotech Institute, Kyung Hee University, Yongin, Republic of Korea; ^3^ Division of Life Science, Plant Molecular Biology and Biotechnology Research Center, Gyeongsang National University, Jinju, Republic of Korea; ^4^ Extension Service Bureau, Rural Development Administration, Jeonju, Republic of Korea; ^5^ National Institute of Agricultural Science, Rural Development Administration, Jeonju, Republic of Korea; ^6^ National Institute of Crop Science, Rural Development Administration, Suwon, Republic of Korea

**Keywords:** endosperm, floury opaque mutant, fructose-6-phosphate 2-kinase/fructose-2, 6-bisphosphatase, rice flour, Seolgaeng, starch

## Abstract

‘Seolgaeng’, an opaque-endosperm rice (*Oryza sativa*) mutant, is used to prepare high-quality dry-milled rice flour. The mutation causing its opaque-endosperm phenotype was unknown. Map-based cloning identified a missense mutation in the gene *FRUCTOSE-6-PHOSPHATE 2-KINASE/FRUCTOSE-2,6-BISPHOSPHATASE 2* (*OsF2KP2*) in Seolgaeng. Transfer DNA insertion and clustered regularly interspaced short palindromic repeat (CRISPR)/CRISPR-associated nuclease 9 (Cas9)-induced *f2kp2* mutants exhibited opaque endosperm. Rice harbors another *F2KP* gene, *OsF2KP1*. CRISPR/Cas9-induced double mutants of *OsF2KP1* and *OsF2KP2* (*f2kp-d*) possessed more opaque endosperm compared to *f2kp2* single mutants, whereas the endosperm of the *f2kp1* single mutant was normal. Grain hardness and damaged starch content were significantly reduced in *f2kp2* mutants compared to the wild type and *f2kp1*. Amylose content was lower than normal in *f2kp2* mutants but not *f2kp1*. Grain hardness and amylose content were much lower in *f2kp-d* than in *f2kp2*. Starch polymerization analysis revealed altered amylopectin structure in *f2kp2* and *f2kp-d* mutants. F2KP activity was lower in *f2kp2* and much lower in the double mutants when compared to the wild types, but *f2kp1* showed no significant difference. In coleoptiles, hypoxia induced *OsF2KP2* expression but downregulated *OsF2KP1*. These results suggest that OsF2KP2 functions as the main F2KP isoform in endosperm experiencing hypoxia, but OsF2KP1 may partially compensate for the absence of OsF2KP2. We propose that F2KP has a crucial role in inorganic pyrophosphate-utilizing energy metabolism for starch biosynthesis in rice endosperm.

## Introduction

Endosperm is the principal storage organ in cereal grains. Flour produced from rice (*Oryza sativa*) endosperm has many culinary uses, including as a gluten-free substitute for wheat flour, and is consumed in processed foods such as cakes, noodles, breads, and confections. Rice varieties vary in their suitability to produce high-quality flour *via* dry milling, which is a relatively simple and low-cost method. Because the main component of milled rice is starch, the functionality of rice flour largely depends on grain hardness and damaged starch content (Patindol et al., 2007).

In contrast to typical translucent rice endosperm, floury opaque endosperm has numerous air spaces that make it easy to mill and thus ideal for rice flour production. This type of endosperm results from the malfunction of amyloplastic starch biosynthetic enzymes, including starch synthases, starch branching enzymes, and starch debranching enzymes. The floury opaque phenotype can also be caused by defects in energy metabolic enzymes that contribute to adenosine triphosphate (ATP) production for starch biosynthesis (Nakamura, 2018; Lee and Jeon, 2020).

The central part of the rice endosperm is subject to hypoxia, which limits ATP production *via* mitochondrial oxidative respiration. The low-oxygen environment necessitates an alternative supply of ATP, derived in this case *via* the inorganic pyrophosphate (PPi)-dependent glycolytic pathway. The first committed step of glycolysis is catalyzed by PPi-dependent fructose-6-phosphate 1-phosphotransferase (PFP), which interconverts fructose-6-phosphate (F6P) and fructose-1,6-bisphosphate (F1,6BP). Rice *PFP* mutants possess a floury opaque endosperm (Duan et al., 2016; Chen et al., 2020). PFP activity is enhanced by fructose-2,6-bisphosphate (F2,6BP), which promotes PPi-dependent glycolytic metabolic flux (Stitt, 1990; Trevanion, 2002; Nielsen et al., 2004). The formation and degradation of F2,6BP is catalyzed by the bifunctional enzyme fructose-6-phosphate 2-kinase/fructose-2,6-bisphosphatase (F2KP). Little is known about the role of F2KP in starch biosynthesis in the endosperm of crop plants, including rice.

The opaque-endosperm rice mutant variety ‘Seolgaeng’ was generated *via* N-methyl-N-nitrosourea treatment. Seolgaeng is commercially used to produce high-quality dry-milled rice flour that exhibits a uniformly fine particle size, with round starch granules and relatively little damaged starch (Kwak et al., 2017). The mutation causing the opaque-endosperm phenotype in Seolgaeng has not been identified. In this study, we discovered using map-based cloning that Seolgaeng harbors a mutation in an *F2KP* gene, *Oryza sativa FRUCTOSE-6-PHOSPHATE 2-KINASE/FRUCTOSE-2,6-BISPHOSPHATASE 2* (*OsF2KP2*). We showed that this mutation is responsible for the floury opaque-endosperm phenotype of Seolgaeng. Our results reveal the crucial role of F2KP in producing F2,6BP, an activator of PPi-dependent PFP, in rice endosperm during starch biosynthesis. In addition, we generated new rice mutants deficient in both *F2KP* isoforms, *OsF2KP1* and *OsF2KP2*, *via* clustered regularly interspaced short palindromic repeat (CRISPR)/CRISPR-associated nuclease 9 (Cas9)-mediated genome editing. The double mutants display improved rice flour characteristics and are more suitable for high-quality dry-milled flour production than Seolgaeng.

## Materials and methods

### Plant materials and growth conditions

Wild-type *japonica* rice (*Oryza sativa*) variety ‘Ilpumbyeo’ (Ilpum) and the opaque-endosperm mutant ‘Seolgaeng’, which was induced by N-methyl-N-nitrosourea treatment of ‘Ilpum’ (Kwak et al., 2017), were used in this study. Wild-type *japonica* rice variety ‘Saeilmi’ was used to produce the mapping population by crossing it with Seolgaeng. Wild-type *japonica* rice variety ‘Taichung’ and its respective transfer DNA (T-DNA) insertion mutant were used for analysis. Wild-type *japonica* rice variety ‘Dongjin’ was used to produce mutant alleles *via* CRISPR/Cas9-mediated genome editing. All plants were grown in the Living Modified Organism paddy fields at the Department of Southern Area Crop Science, Miryang, and Kyung Hee University, Yongin, Korea, under natural conditions during the summer.

### Morphological and physicochemical analysis of rice grains

The ratio of amylose to amylopectin and total starch content were determined using an Amylose–amylopectin Assay kit (K-AMYL; Megazyme, Wicklow, Ireland) and a Total Starch Assay kit (K-TSTA; Megazyme, Wicklow, Ireland), respectively, according to the manufacturer’s instructions. Rice endosperm hardness was determined by measuring the pressure at the grain breakage point using a universal testing machine (Z 0.5, Zwick Roell, Ulm, Germany) (Kwak et al., 2017). To measure damaged starch content, 100 ± 10 mg of rice flour from each mutant and its respective wild type was tested using a Starch Damage Assay kit (K-SDAM; Megazyme, Wicklow, Ireland). For microscopy analysis of the opaque-endosperm phenotype, rice grains were observed on an LED illuminator. Scanning electron microscopy was carried out as described previously (Ryoo et al., 2007). Starch samples coated with gold were observed under a Stereoscan Leica Model 440 instrument (Leica Cambridge) at an accelerating voltage of 20 kV. The chain-length distribution of starch extracted from mature endosperm was determined according to a previously described high performance anion exchange chromatography method (Ryoo et al., 2007).

### Map-based cloning

To develop a mapping population, Seolgaeng was crossed to Saeilmi to yield the line YR29150. The F_2_ individuals from self-fertilized YR29150 plants were used for phenotypic analysis of the endosperm. One of the progeny plants, YR29150-1, an opaque-endosperm line, was backcrossed to Saeilmi to produce a BC_1_F_1_ line named YR32422, which was further propagated to produce BC_1_F_2_ individuals. Kompetitive Allele-Specific PCR (KASP) markers were used to construct an initial map of the causal gene (Cheon et al., 2018). BC_1_F_2_ and BC_1_F_3_ individuals with wild-type endosperm were sequentially analyzed to narrow down the candidate region. To identify mutations in the target interval, genomic DNA sequencing was performed in the Seolgaeng and Ilpum backgrounds.

### Isolation and production of *Osf2kp* mutants

To isolate the mutated DNA in the T-DNA insertional mutant *f2kp2-2*, the primer sets F2KP2-TF/F2KP2-TR and F2KP2-RB/F2KP2-TR (**Supplementary Table S1**) were used to amplify gene-specific and T-DNA-specific regions, respectively. To produce CRISPR/Cas9-mediated single knockout mutants for *OsF2KP1* or *OsF2KP2*, the sequences 5′-AGACACCCCGTGCATCATTG-3′ and 5′-AGTTTGTGGAAGTAATGAGG-3′ were selected as targets, respectively, using the CRISPRdirect program, avoiding off-target effects (Naito et al., 2015). Single-strand oligos for each target were annealed, cloned into the entry vector pOs-sgRNA, and subcloned into the destination vector pH-Ubi-cas9-7 using the Gateway system (Miao et al., 2013). For the *f2kp1 f2kp2* double mutant, tRNA-target-gRNA-tRNA-target-gRNA fragments were synthesized *via* Golden Gate assembly, with 5′-TACTGACATCAGGGATTCCC-3′ and 5′-TGACCTAGCTGCTAGTTGGA-3′ as the respective targets for each gene. The fragments were then cloned into the pRGEB32 vector, which was predigested with *Bsa*I (Xie et al., 2015). The resulting vectors were introduced into wild-type *japonica* rice variety Dongjin by Agrobacterium (*Agrobacterium tumefaciens*)*-*mediated transformation (Jeon et al., 2000). PCR amplicons were generated using gene-specific primers (F2KP1C-seq-F and F2KP1C-seq-R for *OsF2KP1* and F2KP2C-seq-F and F2KP2C-seq-R for *OsF2KP2*) and subjected to Sanger DNA sequencing to genotype the mutants (**Supplementary Table S1**). Finally, CRISPR/Cas9 transgene-free homozygous progeny plants were selected by performing PCR with the vector-specific primers pRGEB32-F and pRGEB32-R and used in subsequent experiments.

### F2KP activity assay

F2KP activity in rice endosperm was determined by measuring the change in activity of PPi-dependent PFP from potato by F2,6BP, which was produced by F2KP, as described previously (Park et al., 2007) with slight modifications. The assay mixture (200 μL total volume) contained 25 mM HEPES (pH 7.5), 1 mM MgCl_2_, 1 mM ATP, 100 mM F-6-P, 1 mM NADH, 0.5 U aldolase, 1 U triose-phosphate isomerase, 1 U glycerophosphate dehydrogenase, and 20 μL crude extract from wild-type and mutant endosperms. A 40-mg sample of developing rice endosperms collected 14 days after pollination was extracted in 1 mL assay buffer. Relative F2KP activity represents the means of three independent experiments. The reaction was started by adding 50 μL 10-mM pyrophosphate. Positive associations between PPi-dependent PFP activity and F2,6BP concentration were determined using F2,6BP (Santa Cruz Biotechnology, Dallas, TX, USA).

### Hypoxia treatment

For the hypoxia stress group, surface-sterilized rice seeds were germinated in water. Twenty seeds were transferred to a 50-mL Corning tube filled with 50 mL sterile water and covered with a lid. For the aerobic (control) group, 20 seeds in a 50-mL Corning tube without a lid were soaked in 1 mL sterile water. Every 12 h, the water was changed for both groups. After a 48-h incubation at a constant temperature of 20°C and a 12-h photoperiod with a uniform light intensity of 150 μmol photons m^–2^ s^–1^, the coleoptiles were harvested from each group for RNA extraction.

### RNA extraction and reverse-transcription quantitative PCR analysis

Total RNA was extracted from the fully developed leaves of 10-week-old plants and coleoptiles of mock- and hypoxia-treated samples using RNAiso Plus (Takara, Japan) and reverse-transcribed into cDNA using a ReverTra Ace qPCR RT Master Mix cDNA Synthesis kit (Toyobo, Japan) according to the manufacturer’s instructions. To measure relative mRNA levels of *OsF2KP1* and *OsF2KP2*, RT-qPCR was performed using Prime Q-Master Mix with SYBR Green I (GeNet Bio, Korea) and a Rotor-Gene 6000 real-time amplification instrument (Corbett Research, Australia). The F2KP1-qRT-F/R and F2KP2-qRT-F/R primer sets were used to examine *OsF2KP1* and *OsF2KP2* transcript levels, respectively. The resulting data were normalized to that of *OsUBQ5* (Os01g0328400) using the primers UBQ5-qRT-F/R (**Supplementary Table S1**).

## Results

### Morphological and physicochemical analysis of Seolgaeng endosperm

**Figure 1:**
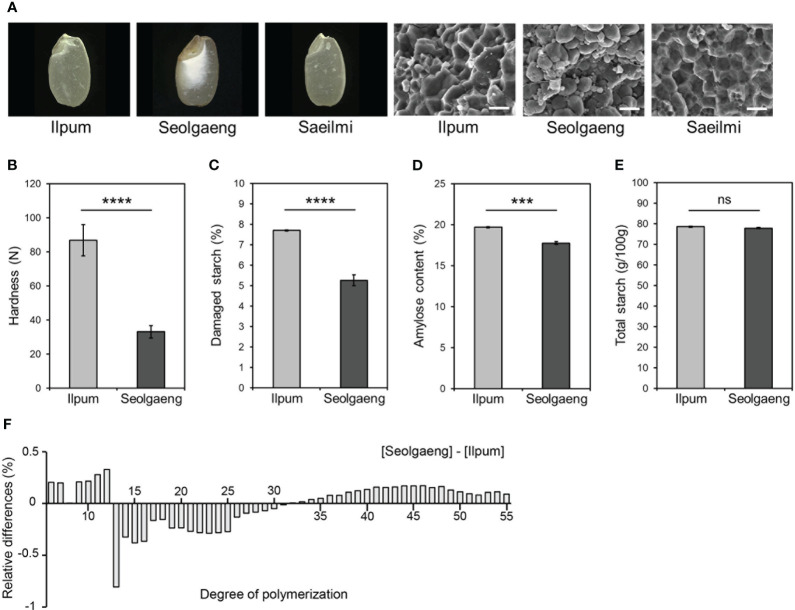
Morphological and physicochemical analysis of Seolgaeng. **(A)** Images of endosperm (left) and scanning electron micrographs of starch granules (right) from three different rice varieties. Scale bars, 50 μm. **(B)** Grain hardness. Data are means ± standard error of the mean (SEM) (*n* = 12). Student’s *t*-tests were used to detect significant differences. ****p < 0.0001. **(C–F)** Comparisons of the starch characteristics of Ilpum and Seolgaeng. **(C)** Damaged starch content. Data are means ± SEM (*n* = 9). ****p < 0.0001. **(D)** Amylose content. Data are means ± SEM (*n* = 4). ***p < 0.001. **(E)** Total starch content. Data are means ± SEM (*n* = 6). ns, not significant. **(F)** Amylopectin chain-length distribution. Relative differences are expressed as the percentage of peak areas of Seolgaeng subtracting Ilpum. Data are the means of three replicates.

Whereas its parental rice variety Ilpum develops a normal endosperm, Seolgaeng, a variety used to produce dry-milled rice flour, is a mutant with a transparent outer surface and an opaque endosperm (Kwak et al., 2017) (**Figure 1A**). Scanning electron microscopy of the endosperm revealed that Ilpum starch granules were polygonal and angular, whereas those of Seolgaeng were round, ellipsoidal, and irregular (**Figure 1A**). Seolgaeng showed significantly lower grain hardness and damaged starch content than Ilpum (**Figures 1B, C**). Amylose content was lower in Seolgaeng compared to Ilpum (**Figure 1D**). Moreover, analysis of the degree of polymerization (DP) of starch revealed that short-chain (DP 6–12) starch production was greater and intermediate-chain (DP 13–30) starch production was less in Seolgaeng compared to Ilpum (**Figure 1F**). These differences likely led to the more rounded starch granules in Seolgaeng. Total starch content was similar in Seolgaeng and Ilpum (**Figure 1E**).

### Map-based cloning of the mutant gene in Seolgaeng

**Figure 2:**
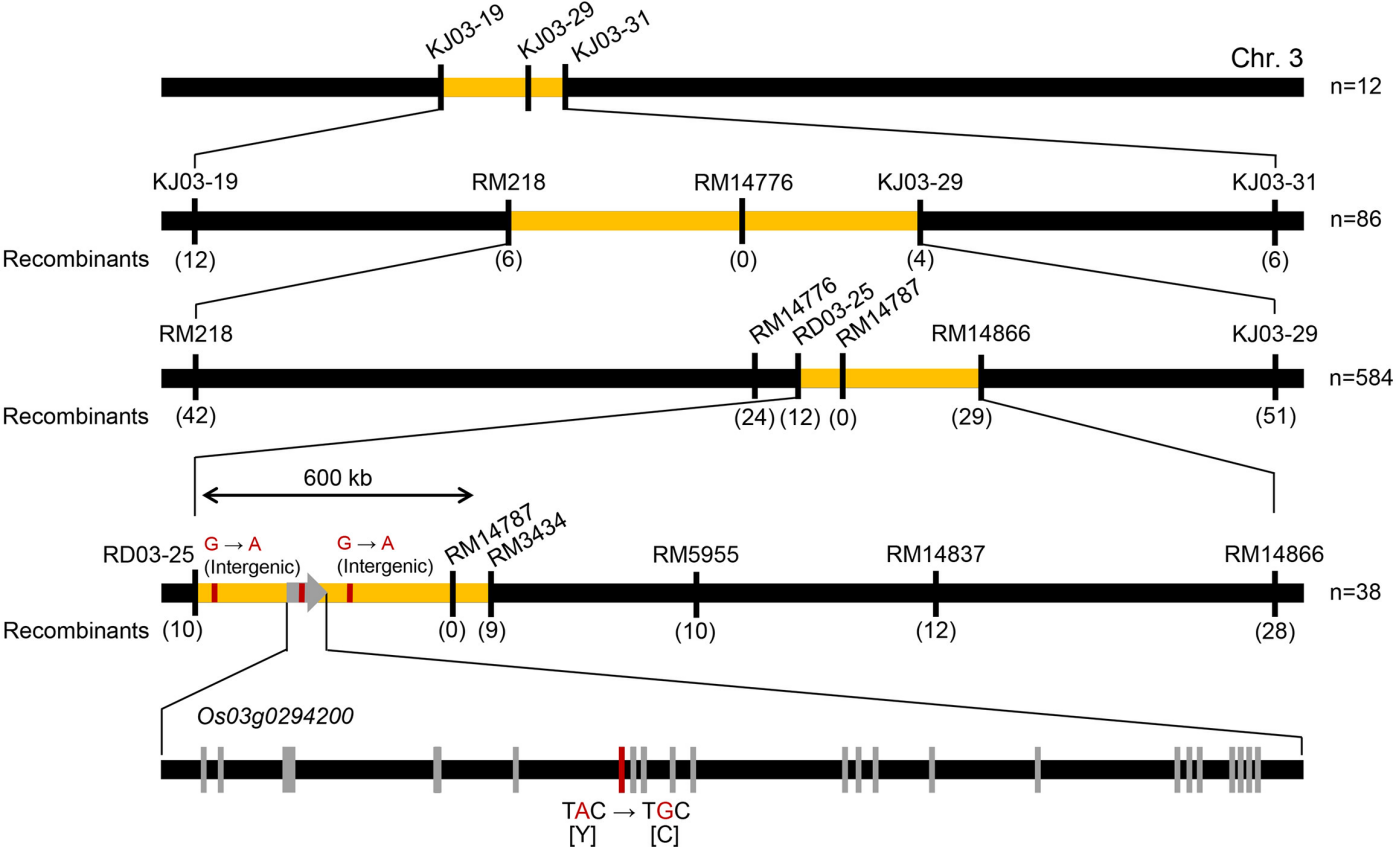
Map-based cloning of the mutated gene in Seolgaeng. The primary map constructed from 12 floury opaque-endosperm individuals located the causal mutation to a 6.6-Mb interval between markers KJ03-019 and KJ03-31 on chromosome 3. The first fine map, constructed from 86 wild-type endosperm individuals, narrowed the candidate region down to a 3.96-Mb interval between markers RM218 and KJ03-29. The second fine map, constructed from 584 wild-type endosperm individuals, identified a 1.6-Mb candidate region between markers RD03-25 and RM14866. The final map, constructed from 38 wild-type endosperm individuals, pinpointed the candidate region to a 600-kb interval between markers RD03-25 and RM3434. Finally, genomic DNA sequencing detected two variations in intergenic regions and one in the sixth exon of Os03g0294200: a nucleotide substitution from A in Ilpum to G in Seolgaeng, which caused an amino acid change at position 266 from tyrosine (Y) to cysteine (C).

To identify the mutation causing the opaque-endosperm phenotype in Seolgaeng, a mapping population was developed from a genetic cross between the mutant Seolgaeng and wild-type Saeilmi (**Figure 1A**). The progeny of self-fertilized F_2_ plants showed a segregation ratio of normal:opaque endosperm of 115:35, which is approximately 3:1 (*X* ^2 = ^0.222; *P* = 0.637). This ratio indicated that a single recessive mutation was responsible for the opaque-endosperm phenotype. We used 153 of 512 available KASP markers to construct an initial map of the causal mutation *via* recessive class analysis by analyzing 12 individuals with floury opaque endosperm. We then sequentially analyzed 86 BC_1_F_2_, 584 BC_1_F_3_, and 38 BC_1_F_3_ backcrossed individuals with wild-type endosperm to narrow down the candidate region. This analysis delineated the causal mutation to a ~600-kb region on chromosome 3 containing 112 annotated genes (**Figure 2**; **Supplementary Table S2**). Genomic DNA sequencing within this region in Seolgaeng and Ilpum detected two intergenic nucleotide changes as well as an A-to-G substitution in the sixth exon of Os03g0294200 in Seolgaeng. This gene encodes OsF2KP2, an enzyme that regulates F2,6BP levels (Park et al., 2007). The A-to-G substitution caused a missense mutation at position 266 of OsF2KP2, from tyrosine (Y) to cysteine (C) (**Figure 2**).

We developed a cleaved amplified polymorphic sequence (CAPS) marker based on the A-to-G change in *OsF2KP2* (**Supplementary Table S3**). The polymorphism associated with the CAPS marker co-segregated perfectly with the wild-type and floury opaque-endosperm phenotypes in F_2_ individuals from a cross between Seolgaeng and Saeilmi (**Supplementary Figure S1A**). In an analysis of 210 floury opaque-endosperm genetic resources, only 4 showed a Seolgaeng-specific allelic pattern, all of which were either Seolgaeng or Seolgaeng-derived lines (**Supplementary Figure S1B**). This result indicates that the mutation is unique to Seolgaeng.

### Functional validation of the *Osf2kp2* mutation

**Figure 3:**
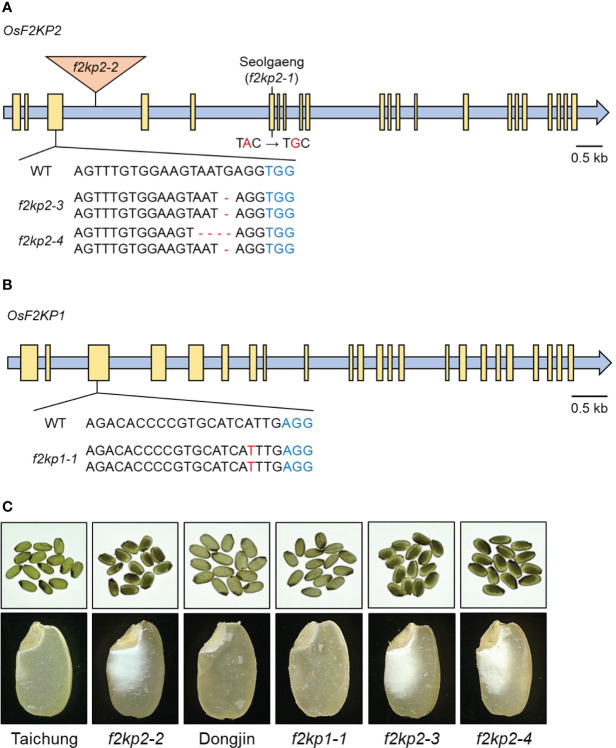
Production of *f2kp1* and *f2kp2* mutants and analysis of their endosperm phenotypes. **(A)** Production of T-DNA insertional and CRISPR/Cas9-edited *f2kp2* mutants. Blue indicates protospacer adjacent motifs and red indicates deletions and substitutions. Triangle, T-DNA insertion site; yellow boxes, exons. **(B)** Production of the CRISPR/Cas9-edited *f2kp1* mutant. Protospacer adjacent motifs and insertion mutations are indicated in blue and red, respectively. Yellow boxes, exons. **(C)** Opaque-endosperm phenotypes (top) and longitudinal endosperm sections (bottom) of *f2kp* mutants compared to the respective wild types.

To determine whether the mutation in *OsF2KP2* is responsible for the opaque endosperm of Seolgaeng, we obtained the T-DNA insertional mutant *f2kp2-2* (variety Taichung) and named the Seolgaeng allele *f2kp2-1* (**Figure 3A**). The expression levels of *OsF2KP2* were reduced in Seolgaeng and *f2kp2-2* compared to the respective wild types (**Supplementary Figure S2A**). The progeny of homozygous *f2kp2-2* mutant plants exhibited opaque endosperm (**Figure 3C**). We created two CRISPR/Cas9-mediated deletion mutants (variety Dongjin) with frameshift mutations in *OsF2KP2*: *f2kp2-3* and *f2kp2-4* (**Figure 3A**). *OsF2KP2* transcript levels were reduced in *f2kp2* mutants (**Supplementary Figure S2A**). The progeny of all homozygous mutants showed opaque endosperm (**Figure 3C**). These results demonstrate that the mutation of *OsF2KP2* leads to opaque endosperm.

### Production and characterization of *f2kp1 f2kp2* double mutants

The rice genome contains a second *F2KP* isoform, *OsF2KP1* (Os05g0164100). Whereas *OsF2KP1* transcripts were abundant in leaves, *OsF2KP2* transcripts were relatively abundant in the endosperm (Park et al., 2007). We generated an *f2kp1* mutant (variety Dongjin) *via* CRISPR/Cas9-mediated genome editing: *f2kp1-1* carried a homozygous T insertion in the third exon of *OsF2KP1*, introducing a frameshift (**Figure 3B**). The expression level of *OsF2KP1* was reduced in *f2kp1-1* (**Supplementary Figure S2B**). Phenotypic analysis revealed that *f2kp1-1* endosperm appeared normal (**Figure 3C**).

**Figure 4:**
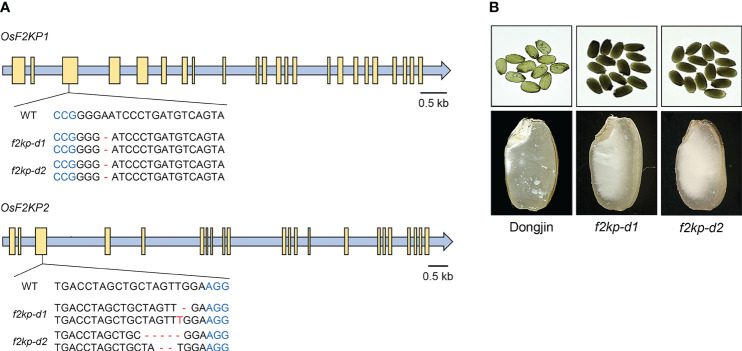
Production of *f2kp* double mutants (*f2kp-d*) and analysis of their endosperm phenotypes. **(A)** Schematic representations of *f2kp-d* mutants generated by CRISPR/Cas9-mediated genome editing. Protospacer adjacent motifs and insertion or deletion (–) mutations are indicated in blue and red, respectively. Yellow boxes, exons. **(B)** Opaque-endosperm phenotypes (top) and longitudinal sections of endosperm (bottom) of *f2kp-d* mutants compared to wild type (Dongjin).

We also generated *f2kp1 f2kp2* double mutants (*f2kp-d*; variety Dongjin) *via* CRISPR/Cas9-mediated genome editing. Double mutants *f2kp-d1* and *f2kp-d2* carried the same homozygous A deletion in *OsF2KP1* and distinct biallelic mutations in *OsF2KP2*, introducing frameshifts in both genes (**Figure 4A**). RT-qPCR analysis confirmed that the expression levels of both *OsF2KP1* and *OsF2KP2* were reduced in *f2kp-d* mutants (**Supplementary Figure S2C**). The endosperms of *f2kp-d* were more opaque than those of *f2kp2* (**Figures 3C**, **4B**).

**Figure 5:**
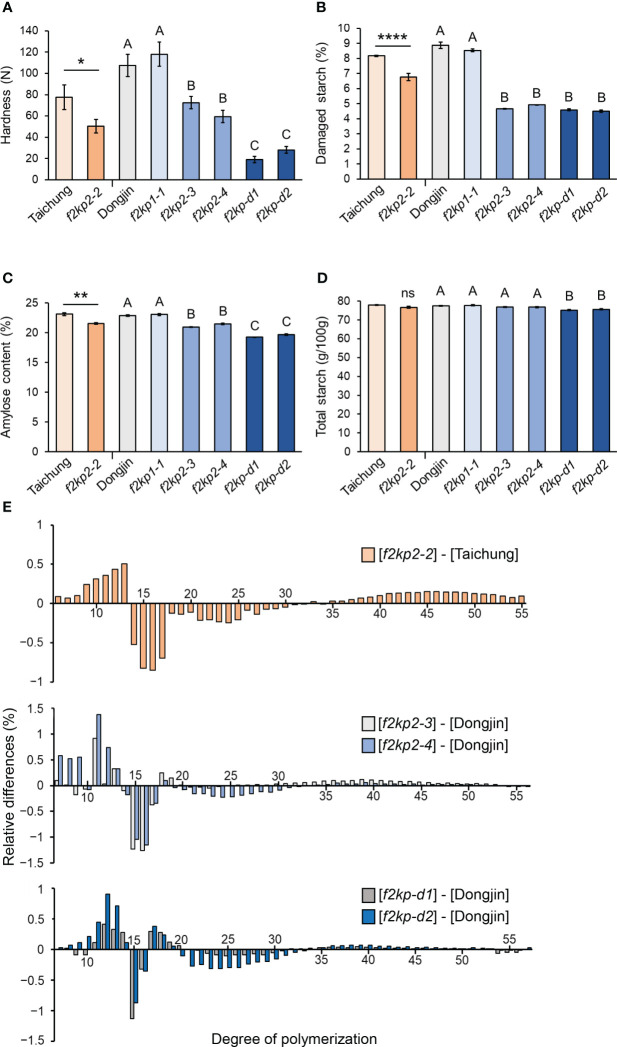
Physicochemical analysis of *f2kp* mutant grains. **(A)** Grain hardness. Data are means ± SEM (*n* = 12). Student’s *t*-tests were used to detect significant differences. *p < 0.05. Different uppercase letters indicate significant differences (*p* < 0.05). **(B)** Damaged starch content. Data are means ± SEM (*n* = 9). ****p < 0.0001. Different uppercase letters indicate significant differences (*p* < 0.05). **(C)** Amylose content. Data are means ± SEM (*n* = 4). **p < 0.01. Different uppercase letters indicate significant differences (*p* < 0.05). **(D)** Total starch content. Data are means ± SEM (*n* = 6). ns, not significant. Different uppercase letters indicate significant differences (*p* < 0.05). **(E)** Amylopectin chain-length distribution. Relative differences are expressed as the percentage of peak areas of mutants subtracting their respective wild types. Data are means of three replications.

Grain hardness and damaged starch content were significantly reduced in *f2kp2* and *f2kp-d* compared to wild-type and *f2kp1* grains (**Figures 5A, B**). Grain hardness was much lower in *f2kp-d* than in *f2kp2*, which is consistent with the severe opaqueness of the double mutant grains (**Figure 4B**). Amylose content was lower in *f2kp2* and much lower in *f2kp-d* compared to the wild types, but *f2kp1-1* showed no significant difference (**Figure 5C**). Analysis of the DP of starch revealed that, as in Seolgaeng, short-chain starch production was higher and intermediate-chain starch production was lower in *f2kp*2 and *f2kp-d* compared to the respective wild types (**Figures 1F**, **5E**). Total starch content did not differ among the *f2kp2* mutants compared to the respective wild types but decreased by 2.9% and 2.4% in *f2kp-d1* and *f2kp-d2*, respectively (**Figure 5D**). These results indicate that changes in amylose content and amylopectin structure resulted in an abnormal starch granule shape in the mutants (Jeon et al., 2010).

### Biochemical analysis of F2KP activity

**Figure 6:**
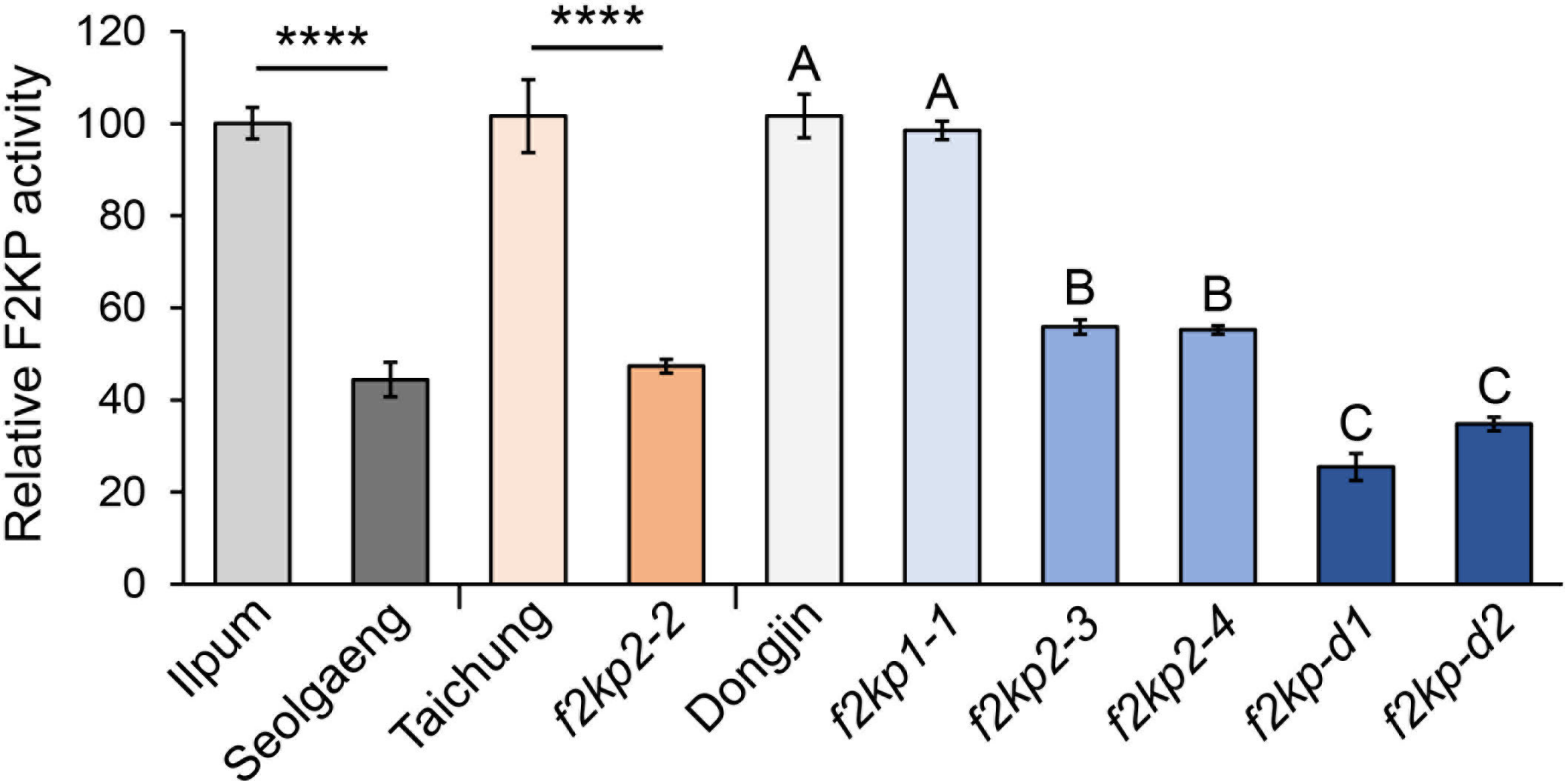
Relative F2KP activity in *f2kp* mutant endosperms. Data are means ± SEM (*n* = 3). Significant differences were analyzed by Student’s *t*-tests. ****p < 0.0001. Different uppercase letters indicate significant differences (*p* < 0.05).

Given that the Y residue at position 266 of OsF2KP2 is conserved in F2KP isoforms from various plant species (**Supplementary Figure S3**), its missense mutation in Seolgaeng likely affects F2KP activity in the endosperm. Enzyme-coupled analysis of F2KP activity in developing endosperms collected 14 days after pollination (when starch biosynthesis is active) revealed that F2KP activity was ~55% lower in Seolgaeng compared to Ilpum (**Figure 6**). F2KP activity was lower in all *f2kp2* mutants and much lower in the double mutants compared to their respective wild types, but *f2kp1-1* showed no significant difference (**Figure 6**). The severity of endosperm opaqueness of the mutants was strongly associated with a reduction in F2KP activity in the endosperm (**Figures 1A**, **3C**, **4B**). These results support the idea that reduced F2KP activity leads to opaque endosperm development.

### Expression analysis of *OsF2KP* genes under hypoxia

**Figure 7:**
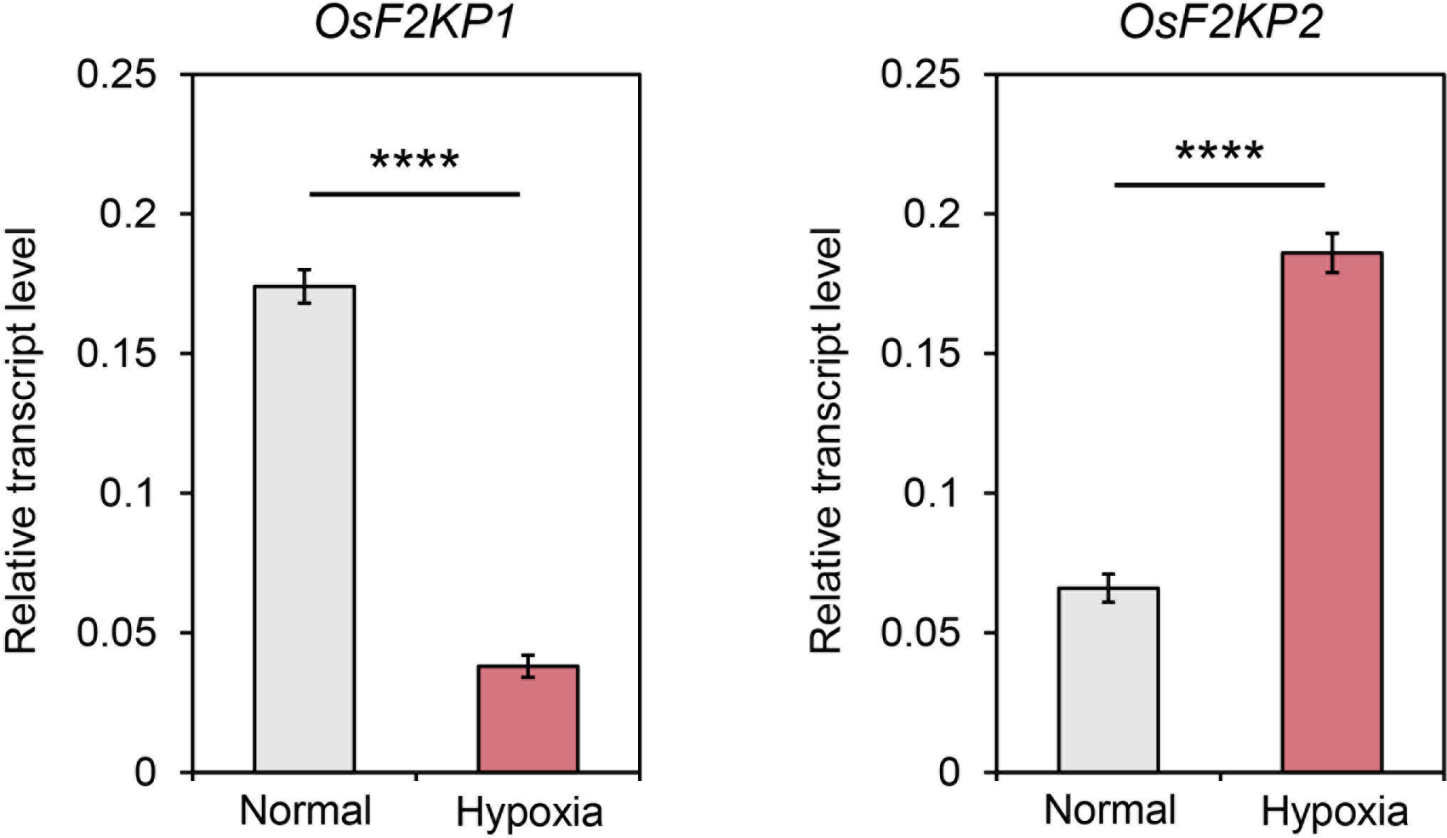
Expression analysis of *OsF2KP1* and *OsF2KP2* in rice coleoptiles under hypoxic conditions. Relative expression was normalized using *OsUBQ5*. Error bars indicate SEM (*n* = 4). Significant differences were analyzed by Student’s *t*-tests. ****p < 0.0001.

PPi-utilizing enzymes can play a crucial role in starch biosynthesis in rice endosperm exposed to hypoxia, a condition in which mitochondrial respiration is limited (Lee and Jeon, 2020). To examine the expression of *OsF2KP1* and *OsF2KP2* in response to hypoxia, we performed RT-qPCR using cDNA produced from total RNA extracted from mock- and hypoxia-treated coleoptile samples. *OsF2KP2* was strongly upregulated under hypoxic conditions, whereas *OsF2KP1* was downregulated (**Figure 7**). These results are consistent with the finding that OsF2KP2 is the main isoform functioning in the central part of the endosperm during hypoxia.

## Discussion

### Impaired F2KP2 function causes floury opaque endosperm in Seolgaeng

Our study provides several lines of evidence that mutations in *OsF2KP2* lead to the development of floury opaque endosperm in Seolgaeng. First, map-based cloning identified a missense mutation in the OsF2KP2 amino acid sequence in Seolgaeng at a residue conserved in F2KPs across plant species. Second, the T-DNA insertional mutant line *f2kp2-2* and additional *f2kp2* mutants created *via* CRISPR/Cas9-mediated genome editing all showed a similar floury opaque-endosperm phenotype. Third, all *f2kp2* mutants showed reduced grain hardness and damaged starch content, low amylose content, and similar changes in amylopectin structure compared to their respective wild types. Last, a biochemical F2KP activity assay confirmed an association between reduced F2KP enzyme activity and the development of floury opaque endosperm. All these data suggest that reduced F2KP function triggers the development of abnormal floury opaque endosperm. Thus, our study demonstrates the crucial function of F2KP in rice endosperm development.

### F2KP functions in PPi-utilizing energy metabolism during endosperm development

**Figure 8:**
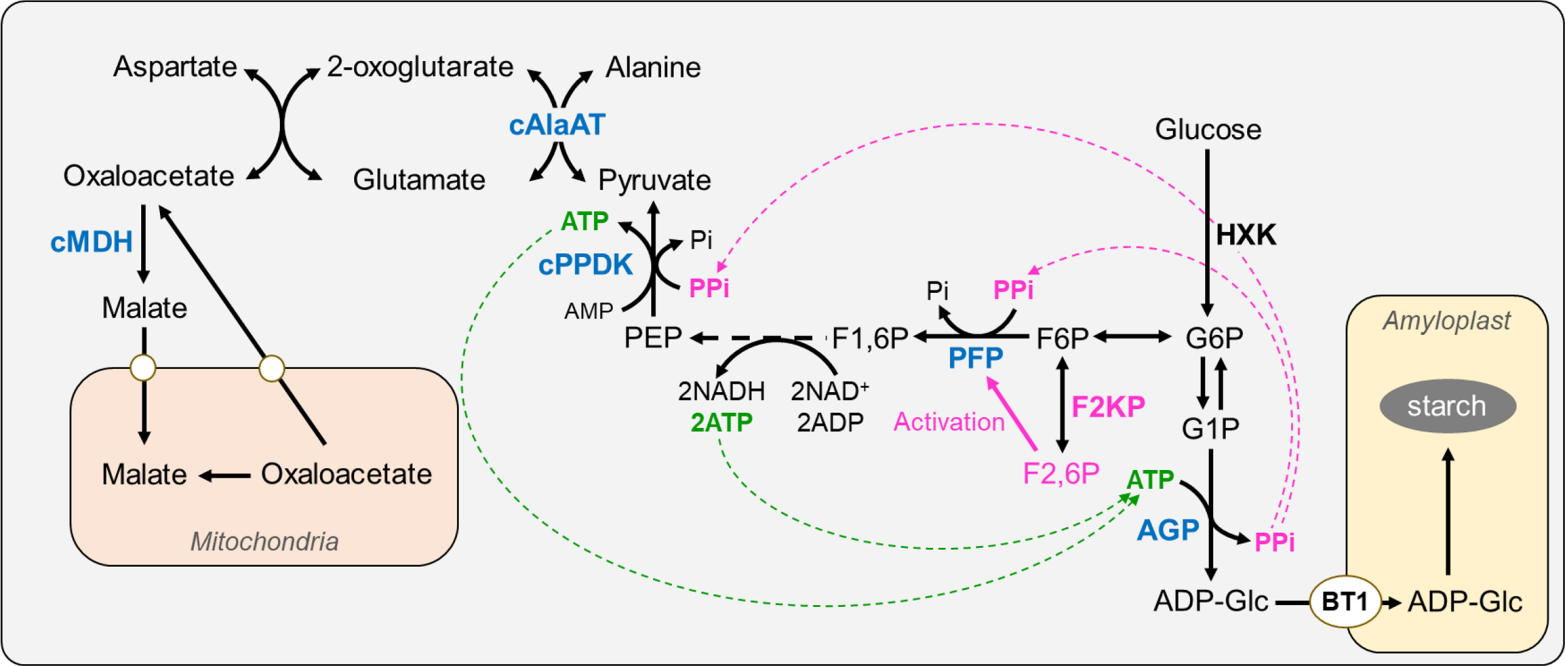
Proposed role of F2KP in rice endosperm starch biosynthesis. The F2KP-related pathway is highlighted in pink. F2KP generates F2,6BP, promoting synthesis of glycolysis-committed F1,6BP, which enters glycolysis. Reduced F2,6BP levels caused by OsF2KP2 deficiency restrict PFP activity, reducing the energy supply for starch biosynthesis. ADP-Glc, ADP-glucose; AGP, ADP-Glc pyrophosphorylase; cAlaAT, cytosolic alanine aminotransferase; BT1, BRITTLE 1 (ADP-glucose transporter); cPPDK, cytosolic pyruvate, orthophosphate dikinase; F1,6P, fructose-1-6-bisphosphate; F2,6P, fructose-2-6-bisphosphate; F2KP, fructose-6-phosphate 2-kinase/fructose-2,6-bisphosphatase; F6P, fructose-6-phosphate; G1P, glucose-1-phosphate; G6P, glucose-6-phosphate; HXK, hexokinase; cMDH, cytosolic malate dehydrogenase; PEP, phosphoenolpyruvate; PFP, PPi-dependent fructose-6-phosphate 1-phosphotransferase.

Rice endosperm is exposed to a hypoxic environment that generates abundant PPi as a byproduct of ADP-Glc pyrophosphorylase in the cytosol. PPi is an alternative energy currency that integrates carbon metabolism from sucrose breakdown with starch biosynthesis in the endosperm (Lee and Jeon, 2020). In this model, PPi-dependent glycolysis *via* PFP and cytosolic pyruvate, orthophosphate dikinase (cPPDK), coupled with the non-cyclic mode of the tricarboxylic acid cycle *via* cytosolic alanine aminotransferase (cAlaAT) and cytosolic malate dehydrogenase (cMDH), can sustain glycolytic flux, which produces adequate ATP levels in the endosperm (**Figure 8**). The malfunction of these enzymes, namely PFP (Duan et al., 2016; Chen et al., 2020), cPPDK (Kang et al., 2005; Lappe et al., 2018; Zhang et al., 2018), cAlaAT (Yang et al., 2015; Zhong et al., 2019), and cMDH (Teng et al., 2019), results in opaque endosperm.

The current study reveals a crucial function of F2KP during endosperm development. F2KP generates F2,6BP, thereby promoting glycolysis-committed F1,6BP production. F2,6BP activates PFP to convert F6P into F1,6BP, which enters glycolysis. Therefore, reduced F2,6BP levels caused by OsF2KP2 deficiency likely restrict PFP activity, reducing the energy supply for starch biosynthesis (**Figure 8**) and resulting in the development of floury opaque endosperm suitable for dry-milled rice flour.

### The *f2kp1 f2kp2* double mutant is an ideal candidate rice variety for high-quality dry-milled rice flour production

Of the two F2KP isoforms, OsF2KP2 is predominant in rice endosperm (Park et al., 2007). Our expression analysis of hypoxia-treated rice coleoptiles suggests that *OsF2KP2* might also be upregulated in the hypoxic central region of rice endosperm. These results are consistent with the floury opaque-endosperm phenotype of all *f2kp2* mutants. Here, we demonstrated that the central region and outer surface of endosperm were more opaque in *f2kp1 f2kp2* double mutant (*f2kp-d*) grains than in the *f2kp2* single mutant. The endosperm of *f2kp1* was indistinguishable from that of wild type. These data suggest that OsF2KP2 functions as the main F2KP isoform in the endosperm, whereas OsF2KP1 may partially compensate for the loss of OsF2KP2. It remains to be determined whether *OsF2KP2* is specifically expressed in the hypoxic central region of the endosperm and *OsF2KP1* in the outer surface.

*f2kp-d* showed reduced grain hardness and amylose content, two favorable characteristics for high-quality dry-milled rice flour. Under normal growth conditions in paddy fields in the summer, *f2kp-d* did not exhibit any marked vegetative growth phenotypes (**Supplementary Figure S4**). However, it will be necessary to observe the growth of the double mutants under stress conditions in the future. Thus, perhaps *f2kp-d* could be used to breed a new rice variety suitable for dry-milled rice flour production.

## Data availability statement

The original contributions presented in the study are included in the article/**Supplementary Material**. Further inquiries can be directed to the corresponding authors.

## Author contributions

J-WK, S-KL, S-HS, DS, J-HC, J-YL, J-MK, HJ, H-MP, and E-KA performed the experiments. J-MK, S-KL, S-HS, J-HL, and J-SJ wrote the manuscript. All authors contributed to the article and approved the submitted version.
