# Supplementary material for: Dry-milled flour rice ‘Seolgaeng’ harbors a mutated fructose-6-phosphate 2-kinase/fructose-2,6-bisphosphatase2
Source: Front Plant Sci. 2023 Aug 10;14:1231914. doi: 10.3389/fpls.2023.1231914 (PMC10449481; doi:10.3389/fpls.2023.1231914)
Supplement: Supplementary file 1 [file Image_1.pdf]

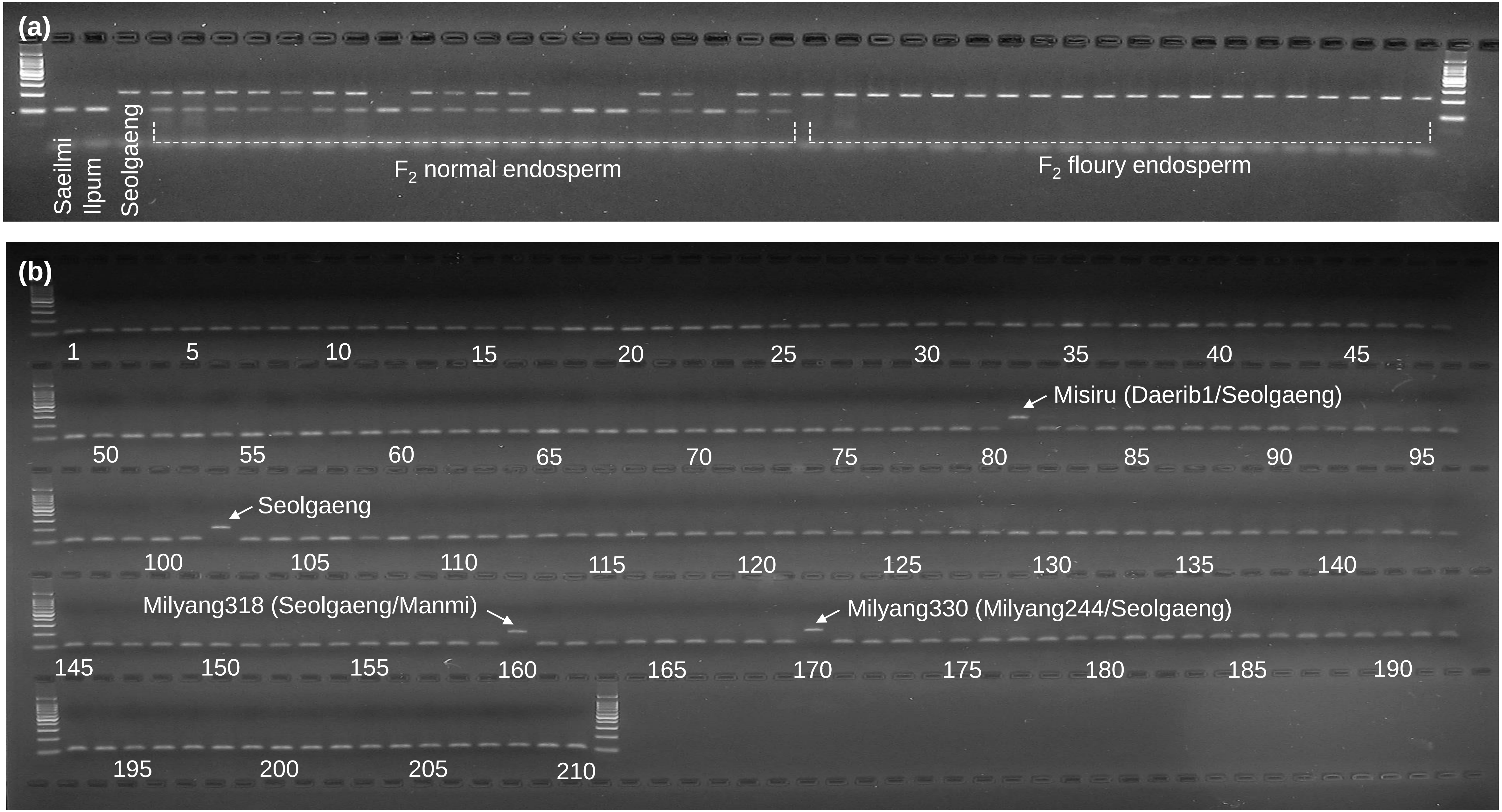

Supplementary Figure S1. Genotypic analysis of *OsF2KP2* alleles using the Seolgaeng-specific CAPS marker. **(a)** Analysis of F_2 progeny derived from genetic cross between Seolgaeng and Saeilmi. **(b)** Analysis of floury opaque endosperm genetic resources.
