# Supplementary material for: Dry-milled flour rice ‘Seolgaeng’ harbors a mutated fructose-6-phosphate 2-kinase/fructose-2,6-bisphosphatase2
Source: Front Plant Sci. 2023 Aug 10;14:1231914. doi: 10.3389/fpls.2023.1231914 (PMC10449481; doi:10.3389/fpls.2023.1231914)
Supplement: Supplementary file 2 [file Image_2.pdf]

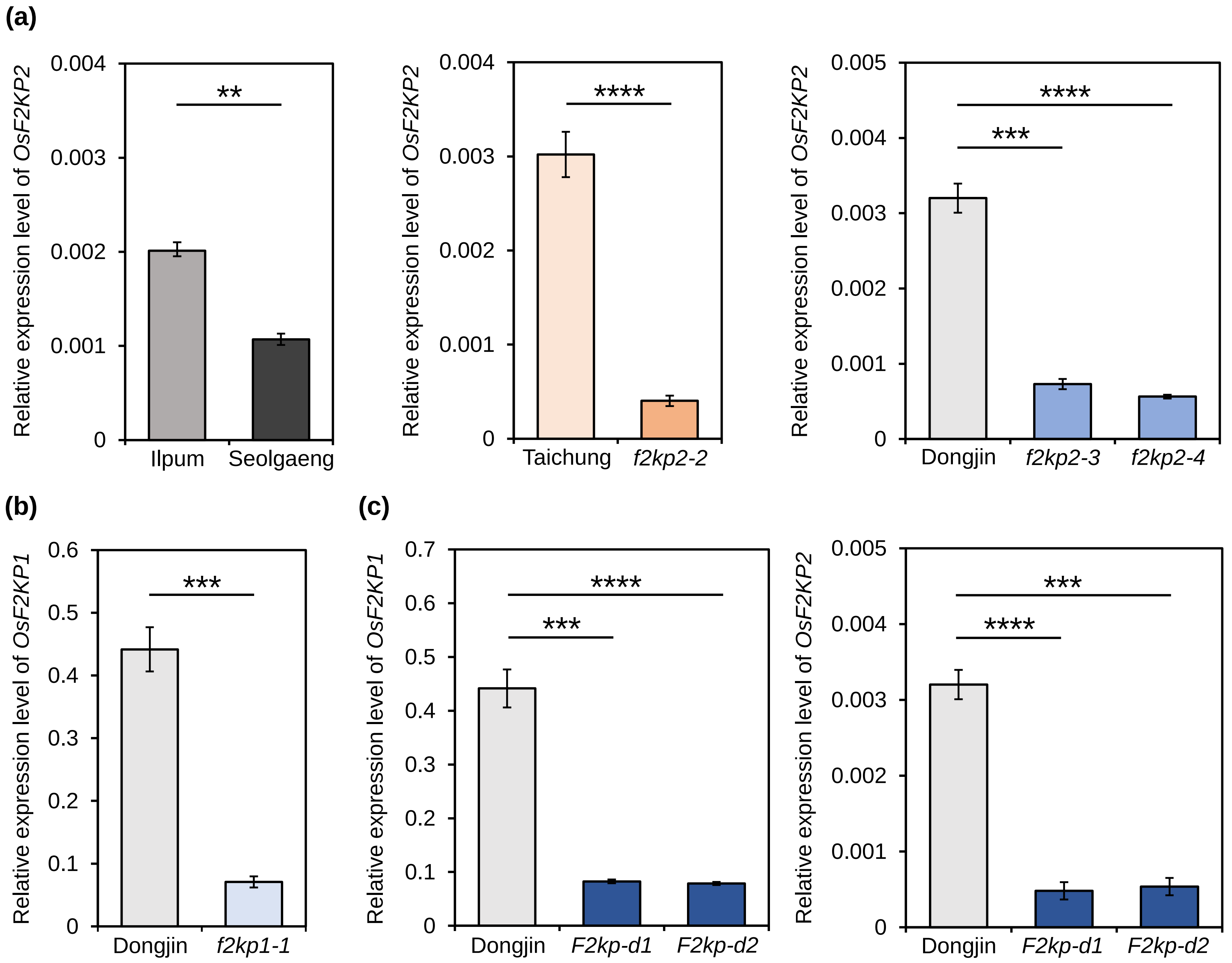

Supplementary Figure S2. Relative transcript levels of *OsF2KP1* and *OsF2KP2* in *f2kp* leaves. Expression level was normalized using *OsUBQ5*. Data are means ± SEM (n=4). **$p < 0.01$; ***$p < 0.001$; ****$p < 0.0001$.
