# Supplementary material for: Dry-milled flour rice ‘Seolgaeng’ harbors a mutated fructose-6-phosphate 2-kinase/fructose-2,6-bisphosphatase2
Source: Front Plant Sci. 2023 Aug 10;14:1231914. doi: 10.3389/fpls.2023.1231914 (PMC10449481; doi:10.3389/fpls.2023.1231914)
Supplement: Supplementary file 3 [file Image_3.pdf]

Supplementary Figure S3. Amino acid sequence alignment of F2KPs from various plant species. The mutation position of Seolgaeng is highlighted in color. Bradi, *Brachypodium distachyon*; GRMZM, *Zea mays*; GSVIVP, *Vitis vinifera*; Os, *Oryza sativa*; Sb, *Sorghum bicolor*.

```
GSVIVP00024699001    ---------------------------------------------MKFLFLI-FKRLPNF    14
LOC_Os03g18310       ----------------------------------MAAAARSSGGMSDQLFVSIKLECPRL    26
Seolgaeng            ----------------------------------MAAAARSSGGMSDQLFVSIKLECPRL    26
Bradi1g65420         -----------------------------------MATAASSGGVPDHLFVSVKLESPRL    25
GRMZM5G824405        --------------------------------------MASSGGISDQLFVSVKLESPHL    22
Sb01g038340          --------------------------------------MASSGGISDQLFVSVKLESPRL    22
GRMZM2G021846        MGTSGSKGIDGVGAVSGVGAGAAGLGGVGAGGDGGEGAAAEAWHGGAQLYVSLKMENALI    60
Sb09g004780          MGTSGSKSIDGVGAVSGVGAAAAGLGG--AGGEGGEGGAAEAWHGGAQLYVSLKMENAEI    58
LOC_Os05g07130       MGTSGSKGIDGVGGVGGGAA-GLGGGEAGDGGGGVGGAASRSWHGGAQLYVSLKMENARI    59
Bradi2g34580         MGTSGSKGMDGVGGVGAAAA-AGGAG--IQDEDGVGGAGMESWHGGAQLYVSLKMENARI    57

GSVIVP00024699001    SSLNMAPTLPCNHPLC----------------------------YLLLLLLLLLKPVSSY    46
LOC_Os03g18310       AEMGLVPHVFGSHPVAGAWDPSKALLMEQEEAALWGLSCIVPSHRETLDFKFLLKPKDNS    86
Seolgaeng            AEMGLVPHVFGSHPVAGAWDPSKALLMEQEEAALWGLSCIVPSHRETLDFKFLLKPKDNS    86
Bradi1g65420         AALVFAPHLFGSHPVAGSWDPSKALPMERVAASIWELSCVLPSQHETLDFKLVLKPKGDS    85
GRMZM5G824405        AELDLAPHLFGSHPVAGSWDPCKALPLERAATAVWEFSCVVPSQHESLDFKFVLKRKGDN    82
Sb01g038340          AELDLAPHLFGSHPVAGSWDPCKALPLERAATSVWEFSCVVPSQHESLDFKFVLKREDDS    82
GRMZM2G021846        -SGDLVPHVYGSEPIIGTWDPSRALAMERELASMWVLSFVVPPDHETLDFKFLLKPKDAE    119
Sb09g004780          -SGDLVPHVYGSEPIIGTWDPSRALAMERELASMWELSFVVPPDHETLDFKFLLKPKDAE    117
LOC_Os05g07130       -TGDLVPHVYGSEPIIGSWDPARALAMERELASMWALSFVVPPDHETLDFKFLLKPKDAD    118
Bradi2g34580         -IGDLVPHVYGSEPIIGSWDPARALAMERELASMWELSFVVPPDHETLYFKFLLKPKDAE    116

GSVIVP00024699001    AQTS---G--KFSLGSSLTAQKNDSFWASPSGDFAFGFQQIGNGGFLLAIWFNKVPEKTI    101
LOC_Os03g18310       SHCIVEEGPDRSLVCGSNEVEIRNALFKFNDETGVVECKIFVETEIL------SPFDLAA    140
Seolgaeng            SHCIVEEGPDRSLVCGSNEVEIRNALFKFNDETGVVECKIFVETEIL------SPFDLAA    140
Bradi1g65420         SECIVEEGPNRQLCCGSNKVEMTGAVFKLSEKD-ELECKVWVETEML------SPFDLAA    138
GRMZM5G824405        PQYIIEEGPNRPLGCQRNEFEMGNALFKLNEGKEVLECKVQVETEML------SPIDLAA    136
Sb01g038340          SQYIIEEGPNRPLGCQSNEFEMRTAVFKLNEGKEVLECKVQIETEML------SPFDLAA    136
GRMZM2G021846        TPCIIEEGPTRLLTGGMLEGDVRVALFKLNGDDEVLEFGVVNKADLV------SPLELAA    173
Sb09g004780          TPCIIEEGPTRLLTGGMLEGDVRVALFKLNGDDEVLEFRVFNKADLV------SPLELAA    171
LOC_Os05g07130       TPCIIEEGPTRHLTGGMLEGDVRIAWFKMNGDHETLEFRVFNKADIV------SPLDLAA    172
Bradi2g34580         TPCVIEEGPTRLLTGGMLEGDVRVANFRLNGDDEVLEFRVFNKADIV------SPLELAA    170

GSVIVP00024699001    IWSANSDNPKPRGSKVE----LTTDGEFI--------LNDQKGKQMWKADLIGPGVAYAA    149
LOC_Os03g18310       SWKAHQEHLQPR-VRGAHDVIMNADSESR-------------------------------    168
Seolgaeng            SWKAHQEHLQPR-VRGAHDVIMNADSESR-------------------------------    168
Bradi1g65420         SWRAHQENLQPSKVRGTYDVVMNAETESRTKNGFASGLELDLEKYVVPTPNMGAGVVYAA    198
GRMZM5G824405        SWRAHQEYFQPSRVRGTHDVTINPGLEGRAKNGFASGLELDLDKYVVPTPNMGSGVVYAA    196
Sb01g038340          SWRAHQEYFQPSRVRGTHDVTINPGLEGRAKNGFASGLELDLEKYVVPTPNMGSGVVYAA    196
GRMZM2G021846        SWRVYKENFQPSKVRGIPDISINVTPTNGAEEGSAATLELDLEHYVVPSPPTAPPNGYAA    233
Sb09g004780          SWRVYKENFQPSKVRGIPDISINVAPTNATEEGSAATLELDLEHYVVPSPPTAPPNGYAA    231
LOC_Os05g07130       SWRVFKENFQPSKVRGIPDVSINVAPAHGTEDTSTESLELDLEHYVIPTP-SAATSEYAA    231
Bradi2g34580         SWRVYKENFQPSQVRGIPDVSINVAPAHATEDGSGSSLELDLEHYVVPTP-TAPPTEYAA    229
```

```
GSVIVP00024699001    MLDTGNFVLASQNSTYLWESFNHPTDTILPTQILEQGSKLVARYSETNYSRGRFMFSLQT    209
LOC_Os03g18310       ------------------------------------------------------------    168
Seolgaeng            ------------------------------------------------------------    168
Bradi1g65420         NLTENPRSLLDHG--VSSN-----NDTTKGT--LQNLIKGDD------------------    231
GRMZM5G824405        SLTENPRSLLQTG--SSSN-----NDTTKDI--LHNSTKG--------------------    227
Sb01g038340          NLTENPRSLLQTG--SSSN-----NDTTKDI--LHSSTKG--------------------    227
GRMZM2G021846        NLAATPASVIQTE--ALLT-----NDILLSDG-IQSPSSVSAKLEDCT-N----------    274
Sb09g004780          NLAATPASMIQTG--ASWT-----NDILLSDG-IQSPSSVSAKLEDRSNN----------    273
LOC_Os05g07130       NLAANPASLIQTG--ALWT-----NDMLLSDG-IQSPSSATADFEDHNNC----------    273
Bradi2g34580         NLAATPASLIQPG--ALWT-----NDMLLSDG-IQSPSSASADFRDHSYH----------    271

GSVIVP00024699001    DGNLVLYTTDFPMDSANFAYWESDTVGSGFLVIFNQSGNIYLIGRNGSILNEVLPNKAST    269
LOC_Os03g18310       ------------------------------------------------------------    168
Seolgaeng            ------------------------------------------------------------    168
Bradi1g65420         ----------------------------------------------------------AP    233
GRMZM5G824405        -----------------------D----------------------------------SS    230
Sb01g038340          -----------------------D----------------------------------SS    230
GRMZM2G021846        --------------HKNIEAWATD----------------------------------SS    286
Sb09g004780          --------------KKNIEAWATD----------------------------------SS    285
LOC_Os05g07130       --------------NKDNVALPSD----------------------------------SF    285
Bradi2g34580         --------------NKDIEASVAD----------------------------------SS    283

GSVIVP00024699001    PDFYQRGILEYDGVFRQYVYPKTAGSRAGGWSSLSSFIPENICTAITAGTGSGACGFNSY    329
LOC_Os03g18310       -----------AKGADGRHVPPQEEQRA-------IFVDRGVGSP-EFARPTNETISMSN    209
Seolgaeng            -----------AKGADGRHVPPQEEQRA-------IFVDRGVGSP-EFARPTNETISMSN    209
Bradi1g65420         PNHYA----NTEKGVDGRHAPSQEEPRA-------VFVDRGVGSP-RLSRPTNETISMGN    281
GRMZM5G824405        LNHYV-NTMKST-I--GGHASSLEEQRA-------MFVDRGVGSP-NFARPTMETFSMSN    278
Sb01g038340          SNHYV-NTMKSA-I--GGHAPSLEEQRA-------MFVDRGVGSP-NFARPTKETFSMSN    278
GRMZM2G021846        KKIQNSGLIESKSVGTFTPMQKLDGQKG-------LFVDRGVGSS-MLPKSASACSLASG    338
Sb09g004780          K---NSGLIESKSVGTFTPLQKLDGQKG-------LFVDRGVGSP-MLPKSASACSLASG    334
LOC_Os05g07130       KKLQVSGIVESKSVDTLTTLQKQDGQKG-------LFVDRGVGST-KFGKSSSACSLASG    337
Bradi2g34580         KKLQVCGMVESKSVGTLIPLQKQDRQKG-------LYVDTGVVSP-KLGKSFSACALASG    335

GSVIVP00024699001    CTLGDDQRPYCQCPPGYTFLDPHDQVKGCRQNFFPEICSEGSHETG-EFDFVRM------    382
LOC_Os03g18310       IKFDSEAKDMPAAEGAVAAAAVADQMYGPKEDRKLAIVLVGLPARGKTFTAAKLTRYLRW    269
Seolgaeng            IKFDSEAKDMPAAEGAVAAAAVADQMYGPKEDRKLAIVLVGLPARGKTFTAAKLTRCLRW    269
Bradi1g65420         IKQEFEAKGMPAAEGAVAAAAVADQMYGPKEDRKLVIVLVGLPARGKTFTAAKLTRYLRW    341
GRMZM5G824405        FKLDSESKGMPAAEGAVAAAAVADQMYGPKEDRKLAIVLVGLPARGKTFTAAKLTRYLRW    338
Sb01g038340          FKLDSDSKGMPAAEGAVAAAAVADQMYGPKEDRKLAIVLVGLPARGKTFTAAKLTRYLRW    338
GRMZM2G021846        FSFGSA-KTMPEAAGAVAAAAVADRLHGSKEDRKLAIVLVGLPARGKTFTAVKLTRYLRW    397
Sb09g004780          FSFGSA-KTMPEAAGAVAAAAVADRLHGSKEDRKLAIVLVGLPARGKTFTAVKLTRYLRW    393
LOC_Os05g07130       LNFGTGKQAMPEAAGAVAAAAVADRLHGSKEDRKLAIVLVGLPARGKTFTAAKLTRYLRW    397
Bradi2g34580         LSFAST-KAMPEAAGAVAAAAVADRLHGSKEDRKLAIVLVGLPARGKTFTAVKLTRYLRW    394
```

```
GSVIVP00024699001     ---TNVDWPLSDYDRFQLFT--------EDECRKACLDDCFCAVAIVREGDCWKKKFPLS     431
LOC_Os03g18310        LGHETKHFNVGKYRRLKHGINQTADFFRGDNKEGVEARNEVAALA-MEDMLSWMQEG-GQ     327
Seolgaeng             LGHETKHFNVGKYRRLKHGINQTADFFRGDNKEGVEARNEVAALA-MEDMLSWMQEG-GQ     327
Bradi1g65420          LGHETKHFNVGKYRRLKHGTSQTADFFRGDNKEGVEARNEVAALA-MEDMLSWMVEG-GQ     399
GRMZM5G824405         LGHETKHFNVGKYRRLKHGTNQTADFFRGDNREGVEARNEVAALA-MEDMLSWMQEG-GQ     396
Sb01g038340           LGHETKHFNVGKYRRLKHGTNQTADFFRGDNREGVEARNEVAALA-MEDMLSWMQEG-GQ     396
GRMZM2G021846         LGHETRHFNVGKYRRLKLGANQSADFFRDDNPEGIEARNEVAALA-MEDMIDWMNGG-GQ     455
Sb09g004780           LGHETRHFNVGKYRRLKHGANQPADFFRDDNPEGIEARNEVAALA-MEDMIDWMNGG-GQ     451
LOC_Os05g07130        LGHETRHFNVGKYRRLKHGANQSADFFRDDNPEGIEARNEVAALA-MEDMIDWMHGG-GQ     455
Bradi2g34580          LGHETKHFNVGKYRRLKHGANQSADFFRADNQQGIEARNEVAALA-MEDMIDWMHGG-GQ     452
                                                   *: .       ..*:* :.:   *      .

GSVIVP00024699001     NGRFDSSNGRIALIKVRKDNSTFPLGSEGKDQATLILTGSVLLGSSVLLNILLLLATAMF     491
LOC_Os03g18310        VGIFDATNS-------------------------------TRNRRNMLMKMAEGKCKIIF     356
Seolgaeng             VGIFDATNS-------------------------------TRNRRNMLMKMAEGKCKIIF     356
Bradi1g65420          VGICDATNS-------------------------------TRSRRNMLMKMAEGKCKIIF     428
GRMZM5G824405         VGIFDATNS-------------------------------TRIRRNMLMKMAEGKCKIIF     425
Sb01g038340           VGIFDATNS-------------------------------TRIRRNMLMKMAEGKCKIIF     425
GRMZM2G021846         VGIFDATNS-------------------------------TRKRRYMLMKMAEGNCKIIF     484
Sb09g004780           VGIFDATNS-------------------------------TRKRRYMLMKMAEGNCKIIF     480
LOC_Os05g07130        VGIFDATNS-------------------------------TRKRRYMLMKMAEGNCKIIF     484
Bradi2g34580          VGIFDATNS-------------------------------TRKRRYMLMKMAEGNCKIIF     481
                       *  *::*.                               .     :*:::    .. :*

GSVIVP00024699001     IYRLNQRKPMIDESRLVMLGTNLKRFAYDE---LEEATDGFKDELGTGAFATVYKGTLAH     548
LOC_Os03g18310        LETICNDQNVLERNMRLKVQR---SPDYAEQTDFEAGVRDFKERLA--YYEKVYEPV---     408
Seolgaeng             LETICNDQNVLERNMRLKVQR---SPDYAEQTDFEAGVRDFKERLA--YYEKVYEPV---     408
Bradi1g65420          VETICNDQDVLERNIRLKVQQ---SPDYAEQTDFEAGVRDFKERLA--YYEKVYEPV---     480
GRMZM5G824405         LETLCNDQDVLERNIRLKVQQ---SPDYAEQTDFEAGVQDFKERLT--YYEKVYEPV---     477
Sb01g038340           LETLCNDQDVLERNIRLKVQQ---SPDYAEQTDFEAGVQDFKERLT--YYEKVYEPV---     477
GRMZM2G021846         LETICNDPNIIERNIRLKIQQ---SPDYAEQLDYEAGLEDFKERLI--NYEKVYEPV---     536
Sb09g004780           LETICNDPNIIERNIRLKIQQ---SPDYADQPDYEAGLEDFKERLI--NYEKVYEPV---     532
LOC_Os05g07130        LETICNDPNIIERNVRLKIQQ---SPDYADQPDYETGVRDFKERLA--NYEKVYEPV---     536
Bradi2g34580          LETICNDRNIIERNVRLKIQQ---SPDYADQPDYEAGLQDFLERLT--NYEKVYEPV---     533
                      :  : :   :::..  : :        * :    * .  .* :.*    : .**: .

GSVIVP00024699001     DNGNLVAVKKLDRAVGEGDKQEFEKIVGAIGRTIHKNLVQLLGFCNKGQHRLLVYEFMSN     608
LOC_Os03g18310        EEGS--YVKMIDMVSGTGGQLQINDISGYLPGRI----VFFLVNCHLT------------     450
Seolgaeng             EEGS--YVKMIDMVSGTGGQLQINDISGYLPGRI----VFFLVNCHLT------------     450
Bradi1g65420          EEGS--YIKMIDMVSGNGGQLQINDISGYLPGRI----VFFLVNCHLT------------     522
GRMZM5G824405         EEGS--YIKMIDMVSGKGGQLKINDISGYLPGRI----VFFLVNCHLT------------     519
Sb01g038340           EEGS--YIKMIDMVSGKGGQIKINDISGYLPGRI----VFFLVNCHLT------------     519
GRMZM2G021846         GEGS--YIKMIDMVKGQDGQLQVNNISGYLPGRI----VFFLVNSHLT------------     578
Sb09g004780           GEGS--YIKMIDMVKGQDGQLQVNNISGYLPGRI----VFFLVNSHLT------------     574
LOC_Os05g07130        QEGS--YIKMIDMVKGQGGQLQVNNISGYLPGRI----VFFLVNSHLT------------     578
Bradi2g34580          EEGS--YIKMIDMVKGQGGQLQVNNISGYLPGRI----VFFLVNSHLA------------     575
                       :*.   :* :* . * ..: :.:.* * :   *    * :*   :
```

```
GSVIVP00024699001       GSLATFLFGNSRPSWYKRMEIILGTARGLLYLHEECSIQAIHGDINPQNILLDDS-L-TA    666
LOC_Os03g18310          -------------------------PRPILLTRHGESLDNVRGRIGGDSSLSETGSLYSR    485
Seolgaeng               -------------------------PRPILLTRHGESLDNVRGRIGGDSSLSETGSLYSR    485
Bradi1g65420            -------------------------PRPILLTRHGESLDNVRGRIGGDSSLSEAGGVYSR    557
GRMZM5G824405           -------------------------PRPILLTRHGESMDNVRGRIGGDSSLSEAGELYSR    554
Sb01g038340             -------------------------PRPILLTRHGESMDNVRGRIGGDSSLSEVGELYSR    554
GRMZM2G021846           -------------------------PRPILLTRHGESLHNVRGRVGGDTVLSETGELYAK    613
Sb09g004780             -------------------------PRPILLTRHGESLHNVRGRVGGDTVLSETGELYAK    609
LOC_Os05g07130          -------------------------PRPILLTRHGESLHNVRGRVGGDTVLSEDGELYSK    613
Bradi2g34580            -------------------------PRPILLTRHGESLHNVRGRVGGDTVLSENGELYAK    610
                                                  * :*  :.  *:. ::* :. :. : : . : :

GSVIVP00024699001       RISDFGLAKLLKMDQTG---------TTTGVMGTKGYAAPEWFKKVPITFKVDVYSFGIV    717
LOC_Os03g18310          KLASFI-EKRLASERTASIWTSTLQRSILTAQPIIGFPKIQWRALDEINA----------    534
Seolgaeng               KLASFI-EKRLASERTASIWTSTLQRSILTAQPIIGFPKIQWRALDEINA----------    534
Bradi1g65420            KLASFV-EKRLKSERTASIWTSTLQRTILTAHPIIGFPKIQWRALDEINA----------    606
GRMZM5G824405           KLASFV-EKRLKSERTASIWTSTLQRTILTAHPIIGFPKIQWRALDEINA----------    603
Sb01g038340             KLASFV-EKRLKSERTASIWTSTLQRTILTAHPIIGFPKIQWRALDEINA----------    603
GRMZM2G021846           KLANFI-EKRLKCEKTATIWTSTLQRTILTASPIVGFPKIQWRALDEINS----------    662
Sb09g004780             KLANFI-EKRLKYEKTATIWTSTLQRTILTASPIVGFPKIQWRALDEINS----------    658
LOC_Os05g07130          KLANFI-EKRLKSEKTASIWTSTLQRTILTASPIVGFPKIQWRALDEINS----------    662
Bradi2g34580            KLANFI-EKRLKSEKTATIWTSTLQRTILTATPIVGFPKIQWRALDEINS----------    659
                        :::.*   * *  :::.         :   .    *:   :*     *.

GSVIVP00024699001       LLELIFCRKNFEPEVEDEKQMVLGEWAYDCYKEGKLDLLVGNDQEALDDIKRLEKFVMVA    777
LOC_Os03g18310          ----GICDGMTYDEI---KKIK--PEEYESRSKDKLRYRYPRGESYLDVIQRLEPVIIEL    585
Seolgaeng               ----GICDGMTYDEI---KKIK--PEEYESRSKDKLRYRYPRGESYLDVIQRLEPVIIEL    585
Bradi1g65420            ----GVCDGMTYDEV---KKNK--PEEYESRRKDKLRYRYPRGESYLDVIQRLEPVIIEL    657
GRMZM5G824405           ----GVCDGMTYDEI---KKSK--PEEYESRRKDKLRYRYPRGESYLDVIQRLEPVIIEL    654
Sb01g038340             ----GVCDGMTYDEI---KKSK--PEEYESRRKDKLRYRYPRGESYLDVIQRLEPVIIEL    654
GRMZM2G021846           ----GVCDGMTYEEI---KKIM--PEEFESRKKDKLRYRYPRGESYLDVIQRLEPVIIEL    713
Sb09g004780             ----GVCDGMTYEEI---KKNM--PEEFESRKKDKLRYRYPRGESYLDVIQRLEPVIIEL    709
LOC_Os05g07130          ----GVCDGMTYEEI---KKVM--PEEFESRKKDKLRYRYPRGESYLDVIQRLEPVIIEL    713
Bradi2g34580            ----GVCDGMTYEEI---KKIM--PEEYESRKKDKLRYRYPRGESYLDVIQRLEPVIIEL    710
                             .*      *:   *:       ::.  :.**     ..:. ** *:*** .::

GSVIVP00024699001       ------------------FWCTQ------EDPSQRPTMKTVMKMLEGATEVPVLQ-----    808
LOC_Os03g18310          ERQRAPVVVISHQAVLRALYAYFADKPLEELPNIEIPLHTIIEIQMGVAGVQEKRYKLMD    645
Seolgaeng               ERQRAPVVVISHQAVLRALYAYFADKPLEELPNIEIPLHTIIEIQMGVAGVQEKRYKLMD    645
Bradi1g65420            ERQRAPVVVIAHQAVLRALYAYFADKPLEEVPKIEIPLHTIIEIQMGVSGVEEKRYKLMD    717
GRMZM5G824405           ERQRAPVVVIAHQAVLRALYAYFADKPLEEVPNIEIPLHTIIEIQMGVAGVQEKRYKLMD    714
Sb01g038340             ERQRAPVVVIAHQAVLRALYAYFADKPLEEVPNIEIPLHTIIEIQMGVAGVQEKRYKLMD    714
GRMZM2G021846           ERQRAPVVVISHQAVLRALYAYFADRPLREVPEIEMPLHTIIEIQMGVTGVEEKRYKLMD    773
Sb09g004780             ERQRAPVVVISHQAVLRALYAYFADRPLREVPDIEMPLHTIIEIQMGVLGVEEKRYKLMD    769
LOC_Os05g07130          ERQRAPVVVISHQAVLRALYAYFADRPLREVPDIEMPLHTIIEIQMGVTGVEEKRYKLMD    773
Bradi2g34580            ERQRAPVVVISHQAVLRALYSYFADRPLREVPDMEMPLHTIIEIQMGVTGVEEKRYKLMD    770
                                          ::.        * *. .  ::*::::  *.  *   :

GSVIVP00024699001       -THPH*---          812
LOC_Os03g18310          AVNSTAGI-          653
Seolgaeng               AVNSTAGI-          653
Bradi1g65420            AVNPTAEI*          725
GRMZM5G824405           AIHPTAGL-          722
Sb01g038340             AIHPTAGL-          722
GRMZM2G021846           ---------          773
Sb09g004780             ---------          769
LOC_Os05g07130          *--------          773
Bradi2g34580            *--------          770
```
