# Supplementary figures and images for: Dry-milled flour rice ‘Seolgaeng’ harbors a mutated fructose-6-phosphate 2-kinase/fructose-2,6-bisphosphatase2
Source: Front Plant Sci. 2023 Aug 10;14:1231914. doi: 10.3389/fpls.2023.1231914 (PMC10449481; doi:10.3389/fpls.2023.1231914)

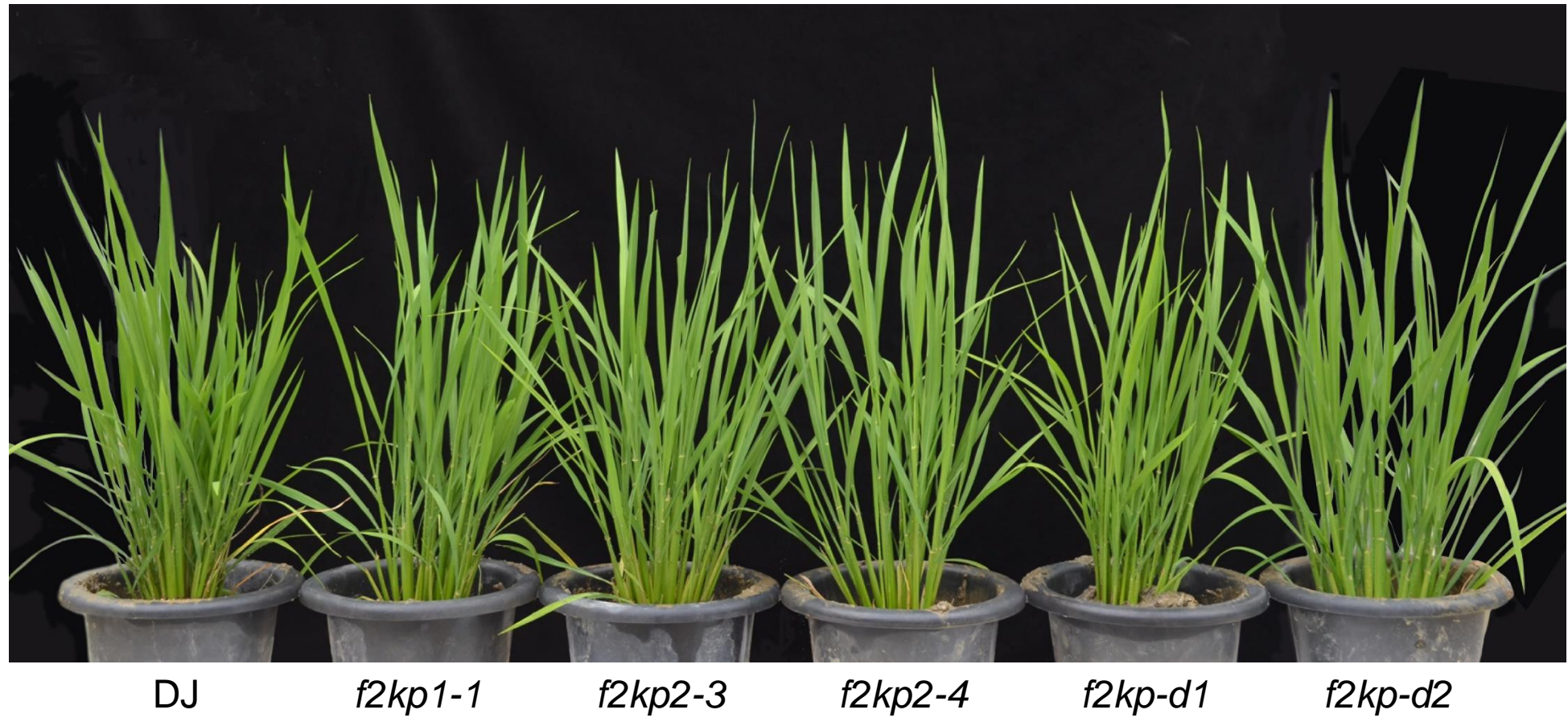

**Supplementary Figure S4.** *f2kp* plants grown in the field for 10 weeks.

Supplement: Supplementary file 4 [file Image_4.pdf]
