# Supplementary material for: Dry-milled flour rice ‘Seolgaeng’ harbors a mutated fructose-6-phosphate 2-kinase/fructose-2,6-bisphosphatase2
Source: Front Plant Sci. 2023 Aug 10;14:1231914. doi: 10.3389/fpls.2023.1231914 (PMC10449481; doi:10.3389/fpls.2023.1231914)
Supplement: Supplementary file 5 [file Table_1.pdf]

Supplementary Table S1. Primers used in this study

Primer	Sequence (5' to 3')
F2KP1C-seq-F	AGCTTGTTTGATGGACTCTATTTA
F2KP1C-seq-R	ACTGAATTGTTTAAGTTTCCCCTA
F2KP2-TF	TATTGAAGCCAAAAGACAATAGCTCACAT
F2KP2-TR	TCGTATTTGCTGCTTAGTTCATTACCATT
F2KP2-RB	ATCCAGACTGAATGCCCACAGG
F2KP2C-seq-F	GTGTAAATACAGAGTTGCAGAGAG
F2KP2C-seq-R	TGTCTCTAGCATTGGATTGTGAAC
pRGEB32-F	CATTTCGTAGTGGGCCATGAAG
pRGEB32-R	TTTACTGTAATTTCTTCTGGCTGG
F2KP1-qRT-F	ATCTTACCTTGATGTGATTCAGAG
F2KP1-qRT-R	ACTCCCATTTGTATCTCAATTATG
F2KP2-qRT-F	AACCTGTAATTATTGAGCTTGAAC
F2KP2-qRT-R	CCATGAGTTTGTATCTTTTCTCTT
UBQ5-qRT-F	GCACAAGCACAAGAAGGTGA
UBQ5-qRT-R	GCCTGCTGGTTGTAGACGTA
