# Supplementary material for: Dry-milled flour rice ‘Seolgaeng’ harbors a mutated fructose-6-phosphate 2-kinase/fructose-2,6-bisphosphatase2
Source: Front Plant Sci. 2023 Aug 10;14:1231914. doi: 10.3389/fpls.2023.1231914 (PMC10449481; doi:10.3389/fpls.2023.1231914)
Supplement: Supplementary file 7 [file Table_3.pdf]

Supplementary Table S3. CAPS marker developed to genotype the Seolgaeng-specific allele of *OsF2KP2*.

Primer	Sequence (5' to 3')	Melting temperature (℃)	GC ratio	Nucleotide length
SG-F	ATTATGCTTCAAATTACCTTTGCAG	66	32.0	25
SG-R	GTGGCTAATACCATGAATATGAGC	68	41.7	24
